# “I’m not a physician, but i’m the expert for my child” experiences of parents caring for their child with a life-limiting condition in an inpatient setting – A qualitative study

**DOI:** 10.1017/S147895152510028X

**Published:** 2025-06-25

**Authors:** Sophie Stößlein, Julia Gramm, Kerstin Karen Hein, Gian Domenico Borasio, Monika Führer

**Affiliations:** Center for Pediatric Palliative Care, Dr. von Hauner Children’s Hospital, LMU Klinikum, Ludwig-Maximilians University, Munich, Germany

**Keywords:** Pediatric palliative care, life-limiting conditions, parents, inpatient, hospital setting

## Abstract

**Objectives:**

Children with life-limiting conditions (LLC) are exposed to frequent hospitalizations, with their parents as indispensable supporters, even in inpatient care. Data on the experiences of parents in a hospital setting are scarce. This study aims to identify the burdens and needs of parents of children with LLC in an inpatient setting to promote family-centered care and thereby strengthen parents as effective partners in care.

**Methods:**

Descriptive qualitative interview study with purposeful sampling and analysis conducted according to the coding method of Kuckartz. A total of 10 interviews with parents (7 mothers, 3 fathers) were included in the analysis.

**Results:**

Main topics reported by parents were (1) structural conditions, (2) commitment and competence of health care professionals, and (3) cooperation between parents and professionals. Parents acquire medical expertise during their child’s illness and learn complex medical procedures to be able to competently care for their child at home. However, their competence is often denied in the inpatient setting. Parents felt that the professionals were overburdened by the complexity of the disease and the fate of the child. They perceived a lack of communication and psychosocial care as burdensome and wished for more psychosocial support and specialized inpatient palliative care structures for their child’s care.

**Significance of results:**

Parents should be supported as equal partners in care to improve the quality of their children’s care. Sole medical care is not enough for children with LLC; therefore, a specialized multidisciplinary palliative care team is highly recommended.

## Introduction

Children with life-limiting conditions (LLC) are exposed to frequent hospitalizations. Parents become indispensable supporters of their care, even in a hospital setting. Regardless of their own workload, parents feel that they must be present at all times as the experts and advocates for their child (Brown and Ritchie 1990; De Geeter et al. 2002). Previous studies on parental experiences show that a hospital stay, especially in an intensive care setting, can be exhausting and burdensome, and that there are several support needs and wishes (Aldridge 2005; Farrell and Frost 1992; Hallström et al. 2002). Thus, special structures for children with LLC are recommended (Bösch et al. 2018). This study aims to identify the burdens and needs of parents of children with LLC in an inpatient setting. Investigating the experiences of parents of terminally ill and hospitalized children can help to better understand their support needs and promote family-centered care. However, so far, little is known about the experiences of inpatient stays from the perspective of parents.

To this end, we interviewed mothers and fathers of children who had been cared for in a dedicated acute pediatric palliative care (PPC) unit about their burdens and needs during hospitalizations, both in the PPC unit and in other pediatric inpatient wards.

## Methods

We conducted a descriptive qualitative interview study with parents who have had inpatient experiences with their child with an LLC on our PPC unit, as well as other hospital wards. Our 8-bed PPC unit offers specialized inpatient care and support for children with LLC and their family caregivers. Most families experienced frequent hospital admissions to both the PPC unit and other pediatric wards (e.g., general inpatient unit, pediatric intensive care unit).

Eligible parents were identified through gatekeepers from the Munich PPC Center according to the above-mentioned criteria.

The method and its reporting follow the Consolidated Criteria for Reporting Qualitative Research (COREQ) (Tong et al. 2007).

### Sampling and recruitment

Parents were contacted by the first author. Of the 13 parents approached, 11 agreed to participate in the study (7 mothers, 4 fathers). Two parents refused to participate, one after their child’s death and the other due to a lack of time. Participants were selected through purposeful sampling if their child had at least one inpatient stay on the PPC unit. To learn about parents’ experiences, we aimed for particularly diverse and information-rich cases. The following factors were varied to reach maximum variation: the child’s age, the child’s illness, the parent’s gender, the admission of the child with or without their parents, and the involvement of a specialized pediatric palliative home care team (SPPHC) prior to admission.

### Data collection

All participants provided written informed consent. The interview was semi-structured according to Helfferich (2005). Four main questions were asked: (1) Can you tell me how your child came to be admitted to the PPC unit? (2) How do you experience everyday life on the PPC unit? (3) What do you feel is good about the PPC unit, and what would you wish for in addition or differently? (4) Do you have experiences with other wards or hospitals? If so, how did you experience the stays there compared to the stay at the PPC unit? Optional supplementary questions were included to obtain more details. At the end of the interview, parents could add further important aspects.

The interview guide was reviewed by a research group with methodological and clinical expertise. Personal interviews were conducted by the first author, S.S., a medical student, from April 2021 to May 2021, partially online via *RedConnect*, a data-secure and certified provider for video consultations, due to pandemic-related contact restrictions. All interviews were audiotaped and transcribed verbatim. All personal data were irretrievably anonymized, and audio recordings were deleted after study completion. This retrospective chart review study involving human participants was in accordance with the ethical standards of the institutional and national research committee and with the 1964 Helsinki Declaration and its later amendments or comparable ethical standards. The ethics committee of the Munich University Hospital reviewed and approved the study protocol and materials (no. 19–2519).

### Data analysis

The interview transcripts were analyzed mainly by S.S. using qualitative content analysis following (Kuckartz 2016) and the software MAXQDA2020. Around 20% of the material was double-coded by J.G. to strengthen reliability. First, deductive main codes were developed starting from the research question. After coding all interviews, the codes were reviewed and discussed by the research group (S.S., J.G., K.H., G.D.B., M.F.). Subcodes were then developed inductively for each main code from the data. For each category, content, coding rules, and prototype examples were defined to increase transparency and reliability. The coding scheme and all codings were reviewed by J.G. and discussed in joint analysis sessions. Then, all relevant text passages were coded with the differentiated coding scheme. Finally, a summary-grid-table was developed. Codings and code summaries were evaluated in thematic order. The interview guideline, the coding list, and the summary-grid-tables are available from the first author (S.S.). In order to maintain anonymity, the participants’ informed consent does not allow us to share the complete interviews with third parties.

## Results

Of the 13 parents contacted, 11 gave their informed consent and were interviewed. One interviewee was excluded from the analysis because he could not remember enough details of his child’s inpatient stay. The characteristics of the remaining 10 participants are shown in Table 1; all patients received palliative care, and no curative treatments were performed in light of the underlying diseases. Admission to the PPC unit occurred mostly due to refractory symptoms in order to achieve symptom control, and in two cases, for postoperative care in a palliative situation. The interviews took between 21 and 86 minutes.

**Table 1.** Parent and child characteristics (10 parents of 10 children)

Parent gender	f = 7, m = 3
Children’s gender	f = 8, m = 2
Children’s age (years)	Median = 5, Range = 0, 9−21 years
Children’s diagnoses	Metachromatic leukodystrophy; Myotonic dystrophy type 1, congenital; Severe brain damage; Cerebral palsy and epilepsy; Spinal muscular atrophy type 1; Niemann–Pick type C; Epileptic encephalopathy; Hypoxic-ischemic encephalopathy; Heart failure with multiple disabilities; Chromosomal abnormality
SPPHC before admission to the PPC unit	Yes = 6, No = 4
First admission to the PPC unit	Yes = 5, No = 5

*Note*: f = female, m = male, Md = median, R = range, PPC = pediatric palliative care, SPPHC = specialized pediatric palliative home care.

Three major topics were reported by the parents: (1) structural conditions, (2) commitment and competence of professionals, and (3) cooperation between parents and professionals. All parents in our study had experiences on a PPC unit as well as on other hospital wards. They reported a strong contrast between their experiences on the PPC unit compared to other hospital wards. At the end of each quote, we mention whether it refers to a general pediatric ward (GPW), an intensive care unit (ICU), or the PPC unit. Parents’ most relevant needs are shown in Table 2.

**Table 2.** Parents’ most relevant needs

Most relevant needs of parents
Being supported in their competencies and being seen as equal partners in care.
At the same time, professionals should take over the responsibility for medical decisions.
Proactive psychosocial support offers to gather strength.
Clear, open, and honest communication.
Competent care for the child.
Having a contact person or an expertise center.
Flexible hospital structures that can adapt to the everyday schedule of the family.
Effective management of medical appointments.

## Structural and organizational conditions

For parents, structural conditions have a great influence on the first impression and the comfort character of a hospital ward.

### Constructional characteristics

Noisy and usually illuminated rooms, poor sleeping facilities for parents, and the lack of recreation rooms or parent rooms were strongly criticized. Staying in shared patient rooms was described as very stressful. Parents highly appreciated a more welcoming and familiar environment with single rooms for patients, a parent’s kitchen, a living and playroom, and balconies to get a breath of fresh air. They preferred spacious, family-friendly rooms that did not reflect the hospital atmosphere and therefore made them feel more comfortable.

When special apartments were available for the parents, they really appreciated this possibility to withdraw, to gain distance, to sleep restfully, and to experience longer time-outs while being available if necessary. Parents embraced the possibility to focus on themselves as parents with their own needs.

She [Daughter] was actually quite relaxed, that’s why [we enjoyed] the parents’ kitchen with the play area, where she spent a lot of time in the end. I think she noticed that she was in a hospital, but she still had her own space where she could move around, and it was not quite so hectic, because the palliative care unit is a bit more to the side, so there is not quite such a hospital feeling. (10_V, referring to the PPC unit)

### Structural obstacles

Poor scheduling management, short-term rescheduling, as well as long waiting times and inflexible processes, were described as stressful. In addition, the family’s daily routine, which is usually determined by the child’s illness, often could not be maintained due to strict hospital routines. Having to adapt to the hospital structure and still take on many of the tasks of care at the same time was described as stressful. Furthermore, admissions without an accompanying adult were hardly possible in most of the pediatric inpatient wards.

Parents were grateful when they were offered specialist consultations directly on the unit. Thus, many concerns could be met in a structured way and the daily routine was more flexible to meet the needs of the child, which was perceived as a great relief.

It was simply great that all the specialists came to the unit for consultations. That was a huge advantage, and it also gave our child a lot of peace and quiet, so that everything could be clarified on the unit. I actually found that to be the decisive advantage of the whole unit. (04_M, referring to the PPC unit)

### Shortage of nursing staff

Parents observed a shortage of nursing staff and, hence, a lack of time for the care of each individual child and even more so for the care of the family. They felt that their child received only medical and nursing care, which was not perceived as sufficient, especially in the context of an LLC. Parents wished for their child to be seen and cared for with age-appropriate needs and developmental tasks despite the illness. Since some parents were used to one-to-one care of their child at home by an outpatient nursing service, they expected the same level of care in the hospital because of their child’s complex care needs. As this was not guaranteed in the hospital, they felt less supported and more stressed than in their domestic care setting.

### Non-medical offers

Although parents appreciated the existing possibilities, such as pedagogical offers and additional therapists for the sick child, some still wished for more non-medical support. When the affected child, together with the whole family, was the focus of care, it was perceived as beneficial.

Well, there is the whole multidisciplinary team. It starts with the nurses, even the ward assistant, if I had any need, […], there are also therapeutic possibilities for me, for example, where I can go […], if the nursing care is good enough at that moment, that I can also accept a therapeutic offer. (5_M, referring to the PPC unit)

## Commitment and competence of care professionals

From the parents’ reports, the attitude and behavior of the professionals and their commitment in taking care of children and families is a central issue.

### Professionals’ commitment

A high commitment to the child’s wellbeing, personal motivation, and empathy, especially among the nurses, were described as key factors affecting parents’ experiences in the hospital. Parents felt relieved and reassured when there was always someone available with time and an open ear, and their concerns were well understood and taken seriously. A caring and supportive attitude of the staff made it easier for parents to deal with and accept existing shortcomings and difficulties. Parents acknowledged that some professionals wanted to support them even more than the existing structures (staffing ratio, available time, etc.) allowed.

I was mostly touched by the commitment, that people are willing to go to such a ward, to work with such a high emotional burden, and still give their best, […] to do something good for the child, to motivate us and to say, hey, this is a difficult phase you are going through, but we will still try to have a nice day with you. (4_M, referring to the PPC unit)

Many professionals, particularly on general wards, were experienced as overburdened – both professionally due to the medical complexity and emotionally in dealing with the severity of the illness and the fate of the family.

### Competence

Above all, the high competence of the professionals strengthened parents’ trust in the care team. Parents emphasized that competence in dealing with difficult and burdensome situations was crucial. This was particularly important because most inpatient stays took place during a crisis, possibly in a life-threatening or end-of-life situation, and was always related to the question: *Will my child survive this crisis?*

When you are admitted to the ward in an acute situation, you are of course very worried as a parent, you never know whether I will now be in a final situation, whether it is now the time, because when I have a child in palliative care, it is always the first thing I think of... (4_M, referring to the PPC unit)

Parents appreciated the availability of a broad spectrum of specialists, both in the inpatient team itself and in the extended team of consultants. The sense of safety and trust in the team enabled parents to hand over parts of their child’s care, including medical responsibility, to the staff. This generated time for “normal parenting.”

## Cooperation between parents and professionals

A cooperative partnership as well as respectful and honest communication between parents and professionals were described as being particularly important.

### Cooperative partnership

Parents perceive themselves as medical experts for their child and her disease. They therefore appreciate respectful interaction and partnership. They want clinicians to consider their experience and knowledge as important for their child. At the same time, parents wish for guidance as medical lay persons in need of caring for a severely ill child. They want their voice to be heard as part of the decision-making process, but they do not want to be the only decision-makers. They wish to hand over the medical responsibility for their child and the medical decisions to the professionals. When they felt left alone in coming to a decision, they felt insufficiently supported.

Parents know best, you get so tired of that at some point. Of course, it’s nice when you are awarded so much, so much competence, and of course you develop that over the years. But somehow you wish that someone says: and that is what we’re doing now, and that’s it, somehow you just don’t get that at all in the course of the disease, when you have a child with such a rare condition. (01_M, referring to a GPW)

### Communication

Parents complain when open and honest communication is missing.

Well, at least [missing communication] worries you immediately, in terms of the situation, how safe you feel and how safe you feel about your child. […] So above all with [the mother] then it had the effect that at some point, […] that you then also doubt the competence of the professionals. And that’s not good, it should really not be like that, because you trust them and in a certain way you depend on them to make you feel safe. (11_V, referring to an ICU)

During inpatient stays, parents often experienced themselves as advocates for their own child against the professionals. They often had to tell the child’s medical story repeatedly, which was perceived as very stressful. Parents criticized the lack of communication and information transfer within hospital teams as well as with specialists from other departments, between hospitals, or with outpatient health care partners. Therefore, parents emphasized the importance and helpfulness of having a multidisciplinary SPPHC team to assist with the transition from an inpatient stay to the home setting.

## Discussion

This study explores parents’ needs and experiences when their child with an LLC is admitted to a hospital setting. Some of the parents’ responses are in line with published reports (Aoun et al. 2020; Callery 1997; Cramer and Klaus 2014; Gill et al. 2021; Inglin et al. 2011; Kirk et al. 2005; Mattsson et al. 2014; Romaniuk et al. 2014; Seliner et al. 2016; Vasli and Salsali 2014). For instance, in our study, as well as in previous studies, parents criticized the architectural structures of hospitals. Severely ill patients and their families prefer single rooms for privacy and a suitable environment for families. Common spaces for exchange with fellow patients and their families and to avoid loneliness are also mentioned as important (Bertuzzi et al. 2023; Curtis and Northcott 2017; Engler et al. 2020).

Other criticisms of hospital structures and organization, such as the standardized and less individualized care and strict schedules on the wards might be more difficult to meet, as standard procedures and structured processes are a necessary feature of inpatient settings. Graham et al. have shown that parents of chronically ill children describe that their needs “do not fit in the acute care model” (Graham et al. 2009).

Our results show that the care needs of children with complex chronic and life-limiting conditions and their families requires a multiprofessional approach (Verberne et al. 2017). Sundal et al. highlighted the need for so-called “home-like care” in the hospital (Sundal 2024; Sundal and Vatne 2020). This is particularly true for severe and multiply disabled children, who comprise the majority of children with LLC. Pedagogical and psychosocial support should be made available to these families during inpatient stays (Brenner et al. 2018; Cassidy et al. 2023).

Taking care of a child with a LLC is a significant challenge for parents. Parents need to become competent healthcare providers and medical experts to care for their child at home (Lazzarin et al. 2018). However, in a hospital setting, they are often still treated as “lay persons” without medical expertise and need to fit into the predefined hospital structures and adapt to hospital rules, while providing a substantial part of the care because of the lack of staff (Rennick et al. 2019). As mentioned in other studies, parents need support in providing and participating in the care of their children and want to be seen as equal partners with exclusive expert knowledge and be respectfully involved in daily care and decision-making processes (Engler et al. 2020; Lam et al. 2006; Melo et al. 2014; Seliner et al. 2016). Parents see themselves as advocates for their child and perceive this task as exhausting (Fields et al. 2023). At the same time, parents want to hand over the medical responsibility to the professionals (Hagvall et al. 2014). Sharing responsibility for a decision can make the further course of the disease more bearable for parents, as the perceived blame does not rest solely on their own shoulders. This ambivalence in the behavior of parents represents a key result of our study.

Having a child with LLC places an immense burden on the parents – not only in terms of medical care but even more so on an emotional and psychological level, often impacting their own health and well-being (Sevin et al. 2022). Thus, it is crucial to provide support and therapeutic offers not only for the children but for their parents, helping them cope with the emotional challenges and process their child’s illness.

Professionals need to develop an understanding of these partially antagonistic needs of parents to address them in a supportive way. In our study, parents highlighted that their satisfaction with an inpatient stay mainly depended on the attitude of the professionals, which is in agreement with recent reports (Fields et al. 2023; Rico-Mena et al. 2023). Especially on general wards, professionals were perceived as overburdened with the complexity of patients with LLC (Seliner et al., 2015). Engler et al. have shown that parents sometimes even felt that their chronically ill child was deprioritized against acute cases (Engler et al. 2020).

A respectful relationship between professionals and the patients and their families can make challenges in caring for children with LLC more bearable and acceptable (Avis and Reardon 2008; McIntosh and Runciman 2008). Professionals should try to support burdened parents’ competence through active care and supportive offers. Concepts for general and intensive inpatient care need to include how to support parents with their coping and involve them in the patient’s care according to their needs and wishes (Doupnik et al. 2017; Smith et al. 2015; Ygge and Arnetz 2004). Therefore, an extension of this study is planned involving inpatient nurses and other health care professionals to learn about their views and needs and how new care concepts can be successfully implemented.

This study has several limitations. One is the sample selection through gatekeepers, which could have biased our results. In addition, some of the responses may reflect social desirability. Since all parents in our study had experiences with PPC, our results may not be generalizable to parents of children with LLC without the experience of PPC. As patients with oncological diseases make up only a small part of patients in PPC (Bender et al. 2017), no parent of a child suffering from cancer participated in our study. Finally, the monocentric study design limits the generalizability of the results to other health care settings.

To conclude, our data show the complex emotional situation of parents of children with LLC in an inpatient setting. On one side, they justly demand support and respect for their acquired competencies as caregivers and “experts for their child.” On the other hand, they wish for the team to take over responsibility for especially medical decisions and thus ease their emotional burden. Thus, our findings underscore the need for specialized pediatric palliative care teams and structures, especially in tertiary pediatric centers, where most of these patients are cared for. The PPC teams should be available to support the ward teams in meeting the complex care needs and the challenging emotional situations and decision-making processes that arise in the care of children with LLCs and their families (Stoesslein et al. 2023).

## Supporting information

10.1017/S147895152510028X.sm001 Stößlein et al. supplementary material Stößlein et al. supplementary material

## Supplementary material

The supplementary material for this article can be found at https://doi.org/10.1017/S147895152510028X.

## References

[ref1] Aldridge MD (2005) Decreasing parental stress in the pediatric intensive care unit: one unit’s experience. *Critical Care Nurse* 25(6), 40–50. doi:10.4037/ccn2005.25.6.40 16311399

[ref2] Aoun SM, Gill FJ, Phillips MB, et al. (2020) The profile and support needs of parents in paediatric palliative care: comparing cancer and non-cancer groups. *Palliative Care and Social Practice* 14, 2632352420958000. doi:10.1177/2632352420958000 33033802 PMC7525220

[ref3] Avis M and Reardon R (2008) Understanding the views of parents of children with special needs about the nursing care their child receives when in hospital: a qualitative study. *Journal of Child Health Care* 12(1), 7–17. doi:10.1177/1367493507085615 18287181

[ref4] Bender H, Riester M, Borasio G, et al. (2017) “Let’s bring her home first.” patient characteristics and place of death in specialized pediatric palliative home care. *Journal of Pain and Symptom Management* 54(2), 159–166. doi:10.1016/j.jpainsymman.2017.04.006. 28602938

[ref5] Bertuzzi A, Martin A, Clarke N, et al. (2023) Clinical, humanistic and economic outcomes, including experiencing of patient safety events, associated with admitting patients to single rooms compared with shared accommodation for acute hospital admissions: a systematic review and narrative synthesis. *BMJ Open* 13(5), e068932. doi:10.1136/bmjopen-2022-068932 PMC10163491 37147093

[ref6] Bösch A, Wager J, Zernikow B, et al. (2018) Life-limiting conditions at a university pediatric tertiary care center: A cross-sectional study. *Journal of Palliative Medicine* 21(2), 169–176. doi:10.1089/jpm.2017.0020 29297749

[ref7] Brenner M, Kidston C, Hilliard C, et al. (2018) Children’s complex care needs: a systematic concept analysis of multidisciplinary language. *European Journal of Pediatrics* 177(11), 1641–1652. doi:10.1007/s00431-018-3216-9 30091109

[ref8] Brown J and Ritchie JA (1990) Nurses’ perceptions of parent and nurse roles in caring for hospitalized children. *Children’s Health Care* 19(1), 28–36. doi:10.1207/s15326888chc1901_4 10106396

[ref9] Callery P (1997) Maternal knowledge and professional knowledge: co-operation and conflict in the care of sick children. *International Journal of Nursing Studies* 34(1), 27–34. doi:10.1016/S0020-7489(96)00033-8 9055118

[ref10] Cassidy L, Quirke MB, Alexander D, et al. (2023) Integrated care for children living with complex care needs: an evolutionary concept analysis. *European Journal of Pediatrics* 182(4), 1517–1532. doi:10.1007/s00431-023-04851-2 36780041 PMC9924191

[ref11] Cramer HW and Klaus (2014) *Die Einschätzung Des Pflegerischen Unterstützungsbedarfs Kranker Kinder Und Ihrer Eltern*. Bielefeld: Institut für Pflegewissenschaft an der Universität Bielefeld (IPW)

[ref12] Curtis P and Northcott A (2017) The impact of single and shared rooms on family-centred care in children’s hospitals. *Journal of Clinical Nursing* 26(11–12), 1584–1596. doi:10.1111/jocn.13485. 27487434

[ref13] De Geeter KI, Poppes P and Vlaskamp C (2002) Parents as experts: the position of parents of children with profound multiple disabilities. *Child: Care, Health and Development* 28(6), 443–453. doi:10.1046/j.1365-2214.2002.00294.x 12568473

[ref14] Doupnik SK, Hill D, Palakshappa D, et al. (2017) Parent coping support interventions during acute pediatric hospitalizations: a meta-analysis. *Pediatrics* 140(3), e20164171. doi:10.1542/peds.2016-4171 28818837 PMC5574731

[ref15] Engler J, Gruber D, Engler F, et al. (2020) Parents’ perspectives on hospital care for children and adolescents with life-limiting conditions: a grounded theory analysis of narrative interviews. *Journal of Palliative Medicine* 23(4), 466–474. doi:10.1089/jpm.2019.0245 31730390

[ref16] Farrell MF and Frost C (1992) The most important needs of parents of critically ill children: parents’ perceptions. *Intensive and Critical Care Nursing* 8(3), 130–139. doi:10.1016/0964-3397(92)90019-g 1421958

[ref17] Fields D, Fraser LK, Taylor J, et al. (2023) What does ‘good’ palliative care look like for children and young people? A qualitative study of parents’ experiences and perspectives. *Palliative Medicine* 37(3), 355–371. doi:10.1177/02692163231154300 36825577 PMC10021114

[ref18] Gill FJ, Hashem Z, Stegmann R, et al. (2021) The support needs of parent caregivers of children with a life-limiting illness and approaches used to meet their needs: a scoping review. *Palliative Medicine* 35(1), 76–96. doi:10.1177/0269216320967593 33103579

[ref19] Graham RJ, Pemstein DM and Curley MAQ (2009) Experiencing the pediatric intensive care unit: perspective from parents of children with severe antecedent disabilities*. *Critical Care Medicine* 37(6), 2064–2070. doi:10.1097/CCM.0b013e3181a00578 19384200

[ref20] Hagvall M, Ehnfors M and Anderzén-Carlsson A (2014) Experiences of parenting a child with medical complexity in need of acute hospital care. *Journal of Child Health Care* 20(1), 68–76. doi:10.1177/1367493514551308 25352538

[ref21] Hallström I, Runesson I and Elander G (2002) Observed parental needs during their child’s hospitalization. *Journal of Pediatric Nursing* 17(2), 140–148. doi:10.1053/jpdn.2002.123020 12029609

[ref22] Helfferich C (2005) *Die Qualität Qualitativer Daten: Manual Für Die Durchführung Qualitativer Interviews*. Wiesbaden: VS Verlag für Sozialwissenschaften

[ref23] Inglin S, Hornung R and Bergstraesser E (2011) Palliative care for children and adolescents in Switzerland: a needs analysis across three diagnostic groups. *European Journal of Pediatrics* 170(8), 1031–1038. doi:10.1007/s00431-011-1398-5 21274564

[ref24] Kirk S, Glendinning C and Callery P (2005) Parent or nurse? The experience of being the parent of a technology-dependent child. *Journal of Advanced Nursing* 51(5), 456–464. doi:10.1111/j.1365-2648.2005.03522.x 16098162

[ref25] Kuckartz U (2016) *Qualitative Inhaltsanalyse. Methoden, Praxis, Computer- unterstützung*. Weinheim and Basel: Beltz Juventa.

[ref26] Lam LW, Chang AM and Morrissey J (2006) Parents’ experiences of participation in the care of hospitalised children: a qualitative study. *International Journal of Nursing Studies* 43(5), 535–545. doi:10.1016/j.ijnurstu.2005.07.009 16143333

[ref27] Lazzarin P, Schiavon B, Brugnaro L, et al. (2018) Parents spend an average of nine hours a day providing palliative care for children at home and need to maintain an average of five life-saving devices. *Acta Paediatrica* 107(2), 289–293. doi:10.1111/apa.14098 28944533

[ref28] Mattsson JY, Arman M, Castren M, et al. (2014) Meaning of caring in pediatric intensive care unit from the perspective of parents: a qualitative study. *Journal of Child Health Care* 18(4), 336–345. doi:10.1177/1367493513496667 23939721

[ref29] McIntosh J and Runciman P (2008) Exploring the role of partnership in the home care of children with special health needs: qualitative findings from two service evaluations. *International Journal of Nursing Studies* 45(5), 714–726. doi:10.1016/j.ijnurstu.2006.12.012 17307182

[ref30] Melo EM, Ferreira PL, Lima RA, et al. (2014) The involvement of parents in the healthcare provided to hospitalzed children. *Revista Latino-Americana de Enfermagem* 22(3), 432–439. doi:10.1590/0104-1169.3308.2434 25029054 PMC4292621

[ref31] Rennick JE, St-Sauveur I, Knox AM, et al. (2019) Exploring the experiences of parent caregivers of children with chronic medical complexity during pediatric intensive care unit hospitalization: an interpretive descriptive study. *BMC Pediatrics* 19(1), 272. doi:10.1186/s12887-019-1634-0 31387555 PMC6683527

[ref32] Rico-Mena P, Güeita-Rodríguez J, Martino-Alba R, et al. (2023) Understanding pediatric palliative care within interdisciplinary palliative programs: a qualitative study. *BMC Palliative Care* 22(1), 80. doi:10.1186/s12904-023-01194-5 37355579 PMC10290414

[ref33] Romaniuk D, O’Mara L and Akhtar-Danesh N (2014) Are parents doing what they want to do? Congruency between parents’ actual and desired participation in the care of their hospitalized child. *Issues in Comprehensive Pediatric Nursing* 37(2), 103–121. doi:10.3109/01460862.2014.880532 24499140

[ref34] Seliner B, Latal B and Spirig R (2016) Erleben und Unterstützungsbedarf von Eltern hospitalisierter Kinder mit Mehrfachbehinderung. *Pflege* 29(2), 73–82. doi:10.1024/1012-5302/a000475 26974279

[ref35] Seliner B, Wattinger A and Spirig R (2015) Experiences and needs of parents of hospitalised children with disabilities and the health professionals responsible for the child’s health-care – a systematic review. *Pflege* 28(5), 263–276. doi:10.1024/1012-5302/a000446 26412679

[ref36] Sevin C, Barth M, Wilds A, et al. (2022) An international study of caregiver-reported burden and quality of life in metachromatic leukodystrophy. *Orphanet Journal of Rare Diseases* 17(1), 329. doi:10.1186/s13023-022-02501-8 36056437 PMC9438185

[ref37] Smith J, Swallow V and Coyne I (2015) Involving parents in managing their child’s long-term condition-a concept synthesis of family-centered care and partnership-in-care. *Journal of Pediatric Nursing* 30(1), 143–159. doi:10.1016/j.pedn.2014.10.014 25458112

[ref38] Stoesslein S, Gramm JD, Bender HU, et al. (2023) “More life and more days”-patient and care characteristics in a specialized acute pediatric palliative care inpatient unit. *European Journal of Pediatrics* 182, 1847–1855. doi:10.1007/s00431-023-04813-8 36795188 PMC10167193

[ref39] Sundal H (2024) Home-like care: collaboration between parents and nurses in everyday situations when children are hospitalized. *Journal of Child Health Care* 28(3), 13674935221149778. doi:10.1177/13674935221149778 PMC11457426 36606622

[ref40] Sundal H and Vatne S (2020) Parents’ and nurses’ ideal collaboration in treatment-centered and home-like care of hospitalized preschool children - a qualitative study. *BMC Nursing* 19, 48. doi:10.1186/s12912-020-00445-7 32536810 PMC7285722

[ref41] Tong A, Sainsbury P and Craig J (2007) Consolidated criteria for reporting qualitative research (COREQ): a 32-item checklist for interviews and focus groups. *International Journal for Quality in Health Care* 19(6), 349–357. doi:10.1093/intqhc/mzm042 17872937

[ref42] Vasli P and Salsali M (2014) Parents’ participation in taking care of hospitalized children: a concept analysis with hybrid model. *Iranian Journal of Nursing and Midwifery Research* 19(2), 139–144. 24834082 PMC4020022

[ref43] Verberne L, Schouten-van Meeteren A, Bosman D, et al. (2017) Parental experiences with a paediatric palliative care team: a qualitative study. *Journal of Palliative Medicine* 31, 026921631769268. doi:10.1177/0269216317692682 28659021

[ref44] Ygge BM and Arnetz JE (2004) A study of parental involvement in pediatric hospital care: implications for clinical practice. *Journal of Pediatric Nursing* 19(3), 217–223. doi:10.1016/j.pedn.2004.02.005 15185252

