# Supplementary material for: “I’m not a physician, but i’m the expert for my child” experiences of parents caring for their child with a life-limiting condition in an inpatient setting – A qualitative study
Source: Palliat Support Care. 2025 Jun 25;23:e127. doi: 10.1017/S147895152510028X (PMC13166731; doi:10.1017/S147895152510028X)
Supplement: Stößlein et al. supplementary material [file S147895152510028Xsup001.docx]

**Supplemental Table**

for the paper by Stoesslein et al. “ ‘I’m not a physician, but I’m the expert for my child’ Experiences of parents caring for their child with a life-limiting condition in an inpatient setting – a qualitative study”

| Key results | Topics | Anchor examples |
| --- | --- | --- |
| Structural and organizational conditions | *Constructional characteristics* | *"We were in different wards there, on repeated occasions for several nights, sometimes even two weeks – structurally, in some cases, a real disaster, that you somehow end up in four-bed rooms, some incredibly noisy rooms, both in terms of insulation to the outside and to the hallway, then, as I said, four-bed rooms, then the parents sleep on some kind of cot or a foldable cube. I don't need a luxury bed or a hotel room, that's clear, but maybe … some kind of standard mattress, where you can at least get four or five hours of sleep, and not with three other children. Just these basic structural or surrounding conditions."* (09_V, referring to a GPW)  *"For me, this sense of safety is very important, this feeling of arriving there and knowing I have a room, knowing that I can also have some undist… well, that I have privacy, that I can be undisturbed for a while, but at the same time, I have this medical background."* (05_M, referring to the PPC unit)  *"A palliative care unit is incomparable to any other ward. They simply try to take much more time and to really focus on the child and the family. […] Also, the structural conditions—I think they are in no way comparable to those of an intensive care unit, and I also believe that the attitude of the nurses is fundamentally different. Otherwise, one couldn’t work there at all."* (07_M, referring to the PPC unit) |
|  | *Structural obstacles* | *"Much more attention needs to be paid to the needs of the patients, much more. What’s happening right now is that the focus is on the needs of the structures. At the moment, practically … Sometimes I feel like the sticker, that I have my sticker, is more important than me being there as a person - to put it bluntly. That hospital sticker, where [the daughter], the number, and whatever else is written on it, that is the most important thing. Without it, you are practically nothing. And it just continues like that. You really have to be careful that somehow the human being remains at the center. I feel like that is getting lost more and more in everyday hospital life."* (05_M, referring to a GPW)  *"Yes. And it's just insane, because that week in the children's hospital emergency room with the [the daughter], these procedures—when you're regularly at that hospital, you go crazy, these procedures that just have to be followed because Paper A needs to go to Desk B and then has to be signed by C before I even get to where I need to be with my daughter in an emergency. And then I sit there, afraid that the machine, that the device won't be available if we now have to sit in the emergency room for another two hours. But the people in the emergency room can't do anything for [the daughter]* *anyway – I know that – because they don’t even know her, but I still have to go through this standard process."* (05_M, referring to a GPW)  *"They don’t adapt to us at all. We have to squeeze in and rearrange everything."* (05_M, referring to a GPW)  *"A well-organized ward that focuses on quality of life – or actually, that focuses on the child, on the family – that ensures you achieve the goals you want to achieve during your stay, […] that would be my perfect ward."* (07_M, referring to any hospital admission) |
|  | *Shortage of nursing staff* | *"Normally, if there is a situation where [the daughter]* *is sick—my daughter – and I have a nursing service, so I have intensive care staff at home who take care of [the daughter]* *for eight hours, and I can step out of the situation. I usually can't do that in the palliative care unit, in the hospital, or during rehab."* (05_M, referring to any hospital admission)  *"I basically have to stand by the bed myself, watch over the child, and the nurses are, of course, stretched to their limits because there are difficult cases everywhere. They only come when the pulse oximeter reacts; otherwise, they don’t come. Or the child just lies there for hours without anyone being able to come. They constantly have to weigh up: Which child do I go to now? Which patient needs me most? And they can only go to those where there is an emergency – everyone else just has to endure it. And now there is this feeling, simply: either I stay there, or my child will suffer."* (04_M, referring to an ICU)  *"Either I have to be strict and lose part of my role as a mother, or I sacrifice many of my child's physical functions and remain only a mother. Those are basically the only two options because, unfortunately, there are no others for us."* (07_M, referring to any hospital admission) |
|  | *Non-medical offers* | *"With the nursing staff, I sometimes had the feeling that [...] some of them preferred to talk in the nurses' room instead of being with the child. They thought, ‘Oh, the mom is here,’ or they didn’t even think to offer, ‘Should I stay in the room so you can eat in peace or read a newspaper or something, just to have a little break.’"* (04_M, referring to the PPC unit) |
| Commitment and competence of care professionals | *Professionals’ commitment* | *"What touched me the most was the commitment – the fact that people are willing to work on such a ward, to handle such an intense emotional burden, and still give their best. Many were highly motivated to do something good for the child, to encourage them, and to say, ‘Hey, this is a difficult phase you’re going through, but we will still try to have a good day together.’ [...] And just the fact that we were helped – that someone actually took us seriously and tried to support us – I really appreciated that."* (04_M, referring to the PPC unit)  "*Parents repeatedly experience that regular hospital wards are overwhelmed by such complex cases, that they simply can’t assess or evaluate the child properly because the child can’t speak, and they have to rely entirely on our descriptions."* (04_M, referring to a GPW)  *"But of course, they are overwhelmed. Yes, absolutely. I still remember – compared to when we had the PEG tube placed – when the nurse sat there and asked, ‘So, what’s going on?’ And then you give a quick explanation, and suddenly the nurse is sitting there crying. And you think, ‘Yeah, damn, okay, my child just had surgery, and now I’m here comforting this nurse who is completely overwhelmed by these kinds of diagnoses.’"* (01_M, referring to a GPW) |
|  | *Competence* | *"* *When you are admitted to the ward in an acute situation, you are of course very worried as a parent, you never know whether I will now be in a final situation, whether it is now the time, because when I have a child in palliative care, it is always the first thing I think of... As a mother or father, I always think, okay, this could be the moment when we need to prepare ourselves, especially when everything is so uncertain. And you just think, okay, now … It’s an immense burden. On the one hand, we are aware of it all the time, but it’s completely different when the actual moment arrives. And as a non-medical person, you can’t assess whether this is something minor that can be managed or whether we are now in a degenerative or final process."* (04_M, referring to the PPC unit)  *"At least in that moment, I didn’t have to carry the medical responsibility and constantly watch and observe – knowing that the nurses and doctors had taken over. I really appreciated that."* (04_M, referring to the PPC unit)  *"In these emergency situations, it was just reassuring to know that there were doctors doing everything they could for [the son] – but not beyond what he wouldn’t have wanted. And also that everyone was so caring – that really did me good, this sense of care, of not feeling alone, of feeling understood. And despite everything, they still focused on what was best for [the son]. Because when that works, you just feel better. In situations like these, I feel so helpless because it breaks my heart that I can’t really do anything for [the son]. Yes. And then knowing that there are doctors and nurses who understand what is best for the patient in such situations – I find that very helpful."* (08_M, referring to the PPC unit) |
| Cooperation between parents and professionals | *Cooperative partnership* | *"You have to understand, we parents of children like this—we are at home, and we become mega-managers of something we never imagined we could handle. That means there’s so much interconnected, and we parents are the ones managing it all. And then someone—whether a doctor or someone else—comes along and simply changes something without discussing it with us."* (05_M, referring to the PPC unit)  *"It was just like … I stood there, knowing that my child was not being taken seriously enough, and neither was I. And that’s why my refusal was so strong – I said, no, I will never go to a hospital again."* (02_M, referring to a GPW) |
|  | *Communication* | *“You also get the feeling that it’s more of a ‘them versus us’ situation rather than working together when the patient or the parents start becoming distrustful."* (11_V, referring to an ICU)  *"Having to tell everything from the beginning over and over again is really exhausting—you start wondering, don’t you talk to each other?"* (02_M, referring to the PPC unit)  *"For example, in the pediatric intensive care unit, I don’t even know how many resident physicians were rotating in and out. No idea, if there were six or seven … If a different resident physician is running around every day, there are bound to be tons of opportunities for communication gaps or miscommunication. Someone needs to stay up to date and actually know what’s going on."* (11_V, referring to an ICU)  *"We saw that we ended up in situations with [my daughter] that we never should have been in. Situations that could have been avoided if someone had just listened for a moment or actually taken in what I was saying."* (10_V, referring to the PPC unit) |
